# Nuclear protein in testis carcinoma of the mediastinum: a case report

**DOI:** 10.1186/s13256-017-1328-x

**Published:** 2017-06-09

**Authors:** Gonçalo Boleto, Jeanne-Marie Perotin, Claire Launois, Emmanuelle Uro-Coste, Philippe Birembaut, Sandra Dury, Hervé Vallerand, François Lebargy, Gaëtan Deslée, Juliette Vella-Boucaud

**Affiliations:** 1 Department of Respiratory Medicine, Reims University Hospitals, Reims, France; 2 INSERM UMR-S 903, Reims, France; 3 Department of Anatomy and Cytopathology, Cancer-Oncopole Institute, University of Toulouse, Toulouse, France; 4 Department of Pathology, Maison Blanche Hospital, Reims University Hospitals, Reims, France; 5 EA 4683 Medical and Pharmacological University of Reims, Reims, France; 6 INSERM UMR 1037, Cancer Research Centre, Toulouse, France; 7 Service de Pneumologie, Hôpital Maison Blanche, CHU Reims, 45 rue Cogncaq-Jay, 51092 Reims cedex, France

**Keywords:** NUT carcinoma, Superior vena cava syndrome, Mediastinal neoplasms

## Abstract

**Background:**

Nuclear protein in testis carcinoma is a rare and very aggressive undifferentiated cancer which characteristically arises in the midline of the head, neck, and mediastinum.

**Case presentation:**

We describe the case of a 46-year-old white woman admitted for superior vena cava syndrome revealing a mediastinal tumor. Pathological examination of specimens obtained by mediastinoscopy revealed an undifferentiated tumor with solid growth and positive immunoreactivity for p40 and negative immunoreactivity for cytokeratin markers. Immunohistochemical staining was positive for nuclear protein in testis, allowing the diagnosis of nuclear protein in testis midline carcinoma of the mediastinum.

**Conclusions:**

We present a rare case of mediastinal nuclear protein in testis carcinoma with diagnosis based on nuclear protein in testis protein positivity and atypical immunohistochemical features including p40 positivity and anti-cytokeratin negativity. Physicians must remain aware of the possibility of nuclear protein in testis carcinoma especially in young patients with thoracic symptoms and suspicion of neoplasm.

## Background

Nuclear protein in testis (NUT) carcinoma is a rare and very aggressive undifferentiated cancer, which characteristically arises in the midline of the head, neck, and mediastinum [1] and shows aggressive behavior with early locoregional invasion and distant metastases [2]. Its incidence is unknown and it occurs mainly in adolescents and young adults [3]. Because of the rarity of this condition, no consensus has been reached concerning the optimal treatment strategy. The prognosis remains extremely poor with a 6.7-month median survival and a global survival of 19% within the first 2 years after diagnosis [2]. The pathophysiology involves a rearrangement of the *NUT* gene on chromosome 15q14 with members of the *BRD* gene family (*BRD*4 and *BRD*3) resulting in a *BRD–NUT* fusion product, which decreases histone acetylation and therefore suppresses squamous cell differentiation [4, 5]. Pathological examination may reveal positivity for cytokeratins (CKs) and p63, a squamous basal cell marker, leading to the incorrect diagnosis of squamous cell carcinoma [6]. We report a case of superior vena cava syndrome revealing a NUT carcinoma of the mediastinum.

## Case presentation

**Fig. 1:**
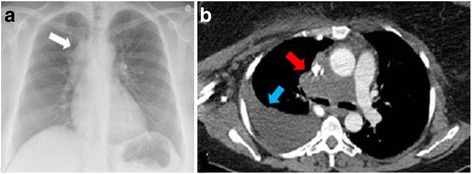
**a** Chest X-ray showing widening of anterosuperior mediastinum (*white arrow*). **b** Contrast-enhanced chest computed tomography scan showing a mediastinal mass with right-sided pleural effusion (*blue arrow*). The mass measures 78 by 40 mm and causes compression of the superior vena cava and pulmonary artery trunk (*red arrow*)

**Fig. 2:**
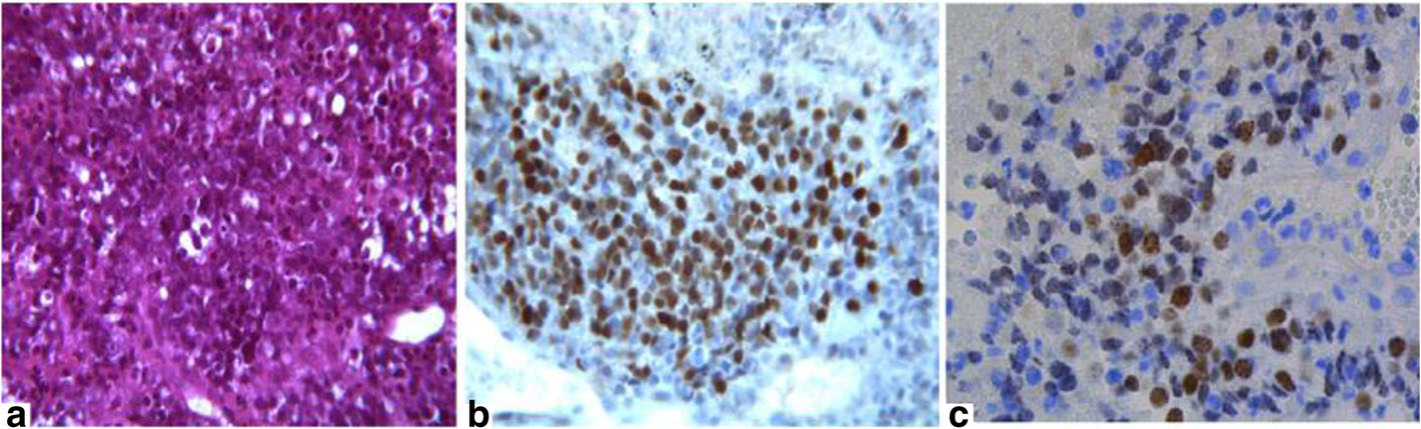
Histomorphological details of nuclear protein in testis carcinoma. **a** The tumor demonstrates a population of cells larger than lymphocytes with a round nucleus, variably prominent nucleoli, with dissociated growth pattern and extensive necrosis; hematoxylin and eosin stain (×400 magnification). **b** Immunohistochemical staining revealed tumor cells diffusely positive for p40 (×400 magnification). **c** Immunohistochemical staining with nuclear protein in testis antibody (×400 magnification) shows nuclear positivity with a speckled pattern

A 46-year-old white woman with no medical history presented to our hospital with complaints of dyspnea, chest pain, dysphagia, cyanosis of the trunk and head, and distended superficial veins over her neck and chest of 3 weeks’ duration; these were all features of superior vena cava obstruction. She had no history of tobacco smoking, alcohol consumption, or illicit substance use. A physical examination showed decreased breathing sounds and dull percussion on the lower lobe of her right lung as well as diffuse wheezing of her right hemithorax. Laboratory tests did not reveal anemia, hydroelectrolytic, or coagulation disorders. A chest X-ray revealed widening of anterosuperior mediastinum (Fig. 1a). A contrast-enhanced chest computed tomography (CT) scan revealed a mediastinal mass with right-sided pleural effusion (Fig. 1b). No evidence of distant metastases was found. Fiberoptic bronchoscopy showed infiltration of the bronchial wall of her carina and her right main bronchus. However, histology of bronchial biopsies did not reveal any tumor infiltration. Mediastinoscopy was therefore performed and histological examination of right laterotracheal lymphadenopathy specimens demonstrated undifferentiated malignant tumor with solid growth composed of cells larger than lymphocytes with a round nucleus, variably prominent nucleoli, with dissociated growth pattern due to the presence of inflammatory cells, polymorphonuclear neutrophils, lymphocytes, and extensive necrosis (Fig. 2). Immunohistochemistry revealed tumor cells diffusely positive for p40 with some reactivity for vimentin. Tumor cells were negative for CK and epithelial membrane antigen (EMA) and for lymphocyte surface markers. Due to these typical findings, subsequent immunohistochemistry for NUT protein was performed and demonstrated marked nuclear positivity (Fig. 2).

After multidisciplinary cancer team meeting discussions, our patient underwent a course of radiation therapy to her chest (20 Gy in five fractions) and received three cycles of carboplatin and paclitaxel. Her symptoms of vena cava syndrome improved and a chest CT scan showed that the size of the mediastinal mass had decreased by 32%: a partial response according to Response Evaluation Criteria in Solid Tumors (RECIST) 1.1 criteria [7]. After the fourth cycle of chemotherapy, she developed severe peripheral neuropathy to paclitaxel leading to discontinuation of treatment. One month after discontinuation of chemotherapy, she developed recurrence of vena cava syndrome symptoms and a chest CT scan showed mediastinal progression of the thoracic mass. Due to her poor general state and despite second-line chemotherapy with carboplatin and pemetrexed, she rapidly progressed to the point of palliative care and died 6 months after the initial diagnosis.

## Discussion

NUT carcinoma is a rare carcinoma with only 39 cases of intrathoracic tumors published to date [3, 8]. Chronic cough, dyspnea, hemoptysis, chest pain, and fatigue are among the main presenting complaints [3].

Histological findings typically show two types of tumor cell populations: (1) poorly differentiated carcinoma, and (2) well-differentiated squamous cell islands with focal keratinization [3, 6]. Positive immunoreactivity to anti-CK antibodies AE1/AE3, as well as EMA (a marker for the epithelial nature of neoplastic cells), p63, and p40 (markers of squamous and basal cell carcinomas) are the usual immunohistochemical findings and should raise the suspicion of NUT carcinoma in young individuals with a midline tumor [6]. The differential diagnosis of mediastinal NUT carcinomas includes undifferentiated malignancies including high-grade hematologic malignancies, endocrine carcinomas, and primitive neuroectodermal tumor [9]. In the case of negative anti-CK antibodies, Ewing sarcoma and small round cell tumors must be ruled out because they are difficult to discriminate morphologically from NUT carcinoma [6].

In our case, immunohistochemistry revealed positivity for p40 and negativity for CK. To the best of our knowledge, our patient is the third reported case of intrathoracic NUT carcinoma with positivity for p40 [3].

Nuclear positivity of more than 50% for anti-NUT antibody with fluorescence *in situ* hybridization (FISH) analysis allows the diagnosis of NUT carcinoma with 100% specificity [9]. Characterization of both fusion genes *BRD4–NUT* and *BRD3–NUT,* and more rarely *NSD3–NUT*, is not mandatory for the diagnosis, but is recommended for its possible association with unique prognostic features [2]. NUT carcinomas lacking *BRD4* fusion gene rearrangements are more differentiated and therefore possibly less aggressive [10]. Fusion gene translocation assessment was not performed in our patient.

At present, there is no consensus concerning the optimal treatment strategy. A combination of multidrug chemotherapy with gemcitabine, docetaxel, and cisplatin and locoregional radiation therapy achieved complete response in a case of NUT midline carcinoma [11]. Administration of histone deacetylase inhibitors such as vorinostat is a promising therapeutic concept, with limitations due to severe side effects [12]. Bromodomain (BRD) and extra-terminal proteins (BET) inhibitors showed rapid antitumor activity in three patients with *BRD4–NUT* fusion NUT carcinoma [13].

## Conclusion

We present a rare case of mediastinal NUT carcinoma revealed by superior vena cava obstruction with a definitive diagnosis based on NUT protein positivity and atypical immunohistochemical features including p40 positivity and anti-CK negativity.
